# Re-evaluation of the role of endoscopic submucosal dissection in the treatment of early gastric cancer based on additional gastrectomy results

**DOI:** 10.1097/MD.0000000000040111

**Published:** 2024-10-11

**Authors:** Dong Won Im, Jae Hun Chung, Dae-Gon Ryu, Cheol Woong Choi, Su Jin Kim, Sun-Hwi Hwang, Si-Hak Lee

**Affiliations:** a Department of Surgery, Pusan National University School of Medicine and Research Institute for Convergence of Biomedical Science and Technology, Pusan National University Yangsan Hospital, Yangsan, Republic of Korea; b Department of Internal Medicine, Pusan National University School of Medicine and Research Institute for Convergence of Biomedical Science and Technology, Pusan National University Yangsan Hospital, Yangsan, Republic of Korea.

**Keywords:** early gastric cancer, endoscopic submucosal dissection, lymph node metastasis, residual tumor, risk factors

## Abstract

Endoscopic submucosal dissection (ESD) plays a pivotal role in treating early gastric cancer (EGC). Some patients require additional gastrectomy because of non-curative ESD. This study aimed to analyze the clinical factors associated with non-curative ESD and to re-evaluate the role of ESD according to its indication criteria. Altogether, 134 patients who had undergone additional gastrectomy with lymphadenectomy for non-curative ESD based on the pathological results of ESD specimens were included. Their data including pre-ESD diagnosis, reasons for requesting additional gastrectomy, and surgical outcomes were analyzed retrospectively. Of the 134 patients with EGC in the final pathology of ESD specimens, 56 underwent staging ESD for a diagnostic approach, of whom 28 were diagnosed with atypical glands and 28 with high-grade dysplasia (HGD) prior to ESD. The remaining 78 patients of the 134 were identified to have EGC and received ESD for therapy. Based on the pathological results of ESD specimens, additional gastrectomy was commissioned with non-curative ESD because of one or more causes such as deep submucosal invasion, lymphatic invasion, positive vertical margin, undifferentiated histology, positive lateral margin, and venous invasion. Regarding surgical specimens, 13 patients had lymph node metastasis (LNM) and 9 had local residual tumor; one of them had both LNM and a local residual tumor. In patients with atypical glands, 4 had LNM and 3 had a local residual tumor; one of them had both LNM and a local residual tumor, and then died of multiple organ metastasis. In patients with HGD, 4 had LNM and 1 had a local residual tumor. Additionally, 4 patients who were absolutely indicated for ESD had LNM, of whom 2 had atypical glands, and the other 2 had HGD. Similarly, in 6 patients with a local residual tumor absolutely indicated for ESD, 2 had atypical glands and 1 had HGD. Positive vertical margin, lymphatic invasion, and deep submucosal invasion were identified as independent risk factors for LNM. ESD may play diagnostic and therapeutic roles in determining the optimal treatment of EGC when the diagnosis is equivocal or insufficient in endoscopic assessments for gastric cancer screening.

## 1. Introduction

Early detection through national cancer screening programs, public awareness on premalignant conditions, and advances in diagnostic techniques have increased the proportion of early gastric cancer (EGC) detected.^[1,2]^

Moreover, endoscopic submucosal dissection (ESD) has become the recommended first option for EGC treatment owing to increased interest in minimally invasive procedures. It preserves the structure of the stomach and its reservoir and digestive functions, thereby reducing the negative impact on nutrient absorption. Therefore, it can provide lower treatment-related complication rates, faster recovery, lower cost, and better quality of life than gastrectomy.^[3]^

However, ESD has clear limitations in the treatment of gastric cancer despite its many advantages. In particular, since ESD focuses only on local treatment of lesions and preserves the mucous, it may be associated with the possibility of development of a local residual tumor, lymph node metastasis (LNM), and the risk of local recurrence. Thus, intensive surveillance should be required and in some cases, additional gastrectomy may be recommended because of non-curative ESD.^[4–6]^

Hence, several studies have been conducted on ESD guidelines, and most endoscopists are currently adopting the indication criteria for the endoscopic treatment of gastric cancer proposed by the Japanese Gastric Cancer Association.^[7]^ However, the factors that were referenced in these indications have been defined and expanded according to the risk of LNM based on the pathological results of surgical specimens from patients with EGC who underwent gastrectomy. Thus, accurately confirming them to be excluded from the criteria by pre-ESD work-up can be difficult.^[8]^ In some clinical situations, the pathologic report via endoscopic forceps biopsy may be ambiguous despite endoscopic macroscopic findings suggesting malignant lesions.^[9,10]^ This discrepancy is frustrating not only for patients, but also for physicians providing treatment as it can lead to overlooking gastric cancer, delaying application of treatment, and increasing costs owing to repeated endoscopic biopsies. Meanwhile, although indications were satisfied in pre-ESD assessments, approximately 32.4% of the pathological results of ESD specimens could occasionally be revealed as non-curative ESD such as beyond the indication, deep submucosal invasion, lymphatic invasion, positive vertical margin, undifferentiated histology, positive lateral margin, and venous invasion.^[11]^ In these cases, additional gastrectomy with lymph node (LN) dissection is required because of the possibility of LNM and local residual tumors.^[12]^ Therefore, this emphasizes the need for precise pretreatment evaluations of gastric lesions. Moreover, reconsidering the validity of applying them to the ESD indication criteria is reasonable.

In the present study, we reviewed surgically resected cases of non-curative ESD in terms of their histopathologic outcomes, as well as the incidence and risk factors of LNM and local residual tumors. In addition, we re-evaluated the recently established indications for ESD and reassessed the role of ESD for gastric lesions of indeterminate pathology based on the indications for the treatment of EGC.

## 2. Methods

### 2.1. Patients

**Figure 1.**
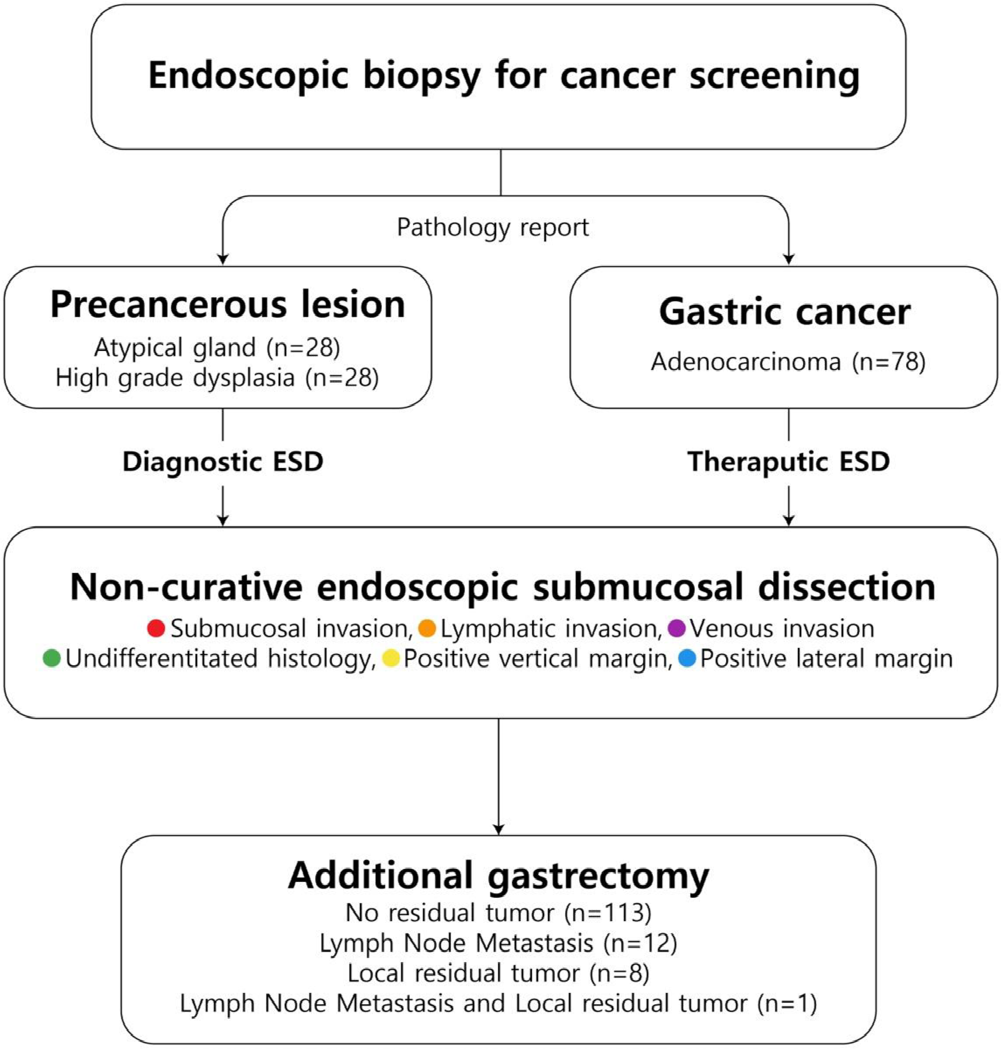
Study flowchart. ESD = endoscopic submucosal dissection.

We reviewed the clinicopathologic data of 134 patients who underwent additional gastrectomy with lymphadenectomy for non-curative ESD based on the pathologic results of ESD specimens that were finally diagnosed as adenocarcinoma at a single academic hospital from January 2012 to December 2022. All patients met the ESD indication criteria for EGC in pre-ESD assessments and underwent en-bloc resection. They were divided into 3 groups according to the pathological results of the surgical specimen as follows: LNM group (n = 13), local residual tumor (n = 9) group, and no residual tumor group (n = 113), which was defined as patients without LNM or local residual tumor in the surgical specimen after additional gastrectomy. One patient had both LNM and a local residual tumor. Data on pre-ESD diagnosis via endoscopic forceps biopsy, reasons for requesting additional gastrectomy, and their surgical outcomes were analyzed retrospectively to identify the risk factors for LNM and local residual tumors, and to reassess the applied ESD indications (Fig. 1). These data have been collected prospectively from the database of patients referred to the Department of Surgery at our institution for non-curative ESD. The ethics committee of our institution approved the study protocol (institutional review board number: 55-2024-013).

Baseline demographics included sex, age, and body mass index. Operation method, operative time, and blood loss were used as variables related to surgery. The postoperative recovery parameters were length of hospital stay, day of first flatus, and day of soft diet initiation.

### 2.2. Definition of non-curative ESD

Non-curative ESD was determined based on histopathologic evaluation of en-bloc resected ESD specimens. It was defined as resection that did not meet the absolute or expanded indications of ESD, or that had deep submucosal invasion, lymphatic invasion, positive vertical margin, undifferentiated histology, positive lateral margin, and venous invasion according to the Japanese Classification of Gastric Carcinoma.^[7]^

### 2.3. Tumor-related pathological variables

Tumor-related pathologic variables were evaluated based on the Japanese Classification of Gastric Carcinoma.^[13]^

Tumor size was measured by pre-ESD endoscopy and pathology of ESD specimen, and the longest diameter of the lesion was measured. Tumor invasion depth was classified into 3 grades as follows: M, mucosal invasion; SM1, minute submucosal invasion (<500 μm from the muscularis mucosae); and SM2, deep submucosal invasion (≥500 μm from the muscularis mucosae). Pre-ESD tumor invasion depth was assessed through conventional white light endoscopy, abdominal computed tomography, and in some cases, endoscopic ultrasonography (EUS). Tumor location was categorized into longitudinal or circular lesions. The location of a longitudinal lesion was classified into the upper, middle, or lower body of the stomach according to the lines connecting the trisected points on the lesser and greater curvatures. For the location of circular lesions, the stomach was divided into 4 equal parts: lesser curvature, greater curvature, anterior wall, and posterior wall. The gross tumor type was grouped as “nondepressed” or “depressed,” and the main gross type was assumed if the tumor was a mixed type. EGC types 0-I, 0-IIa, 0-IIb, 0-IIa + IIb, 0-IIb + IIa, 0-IIa + IIc, and 0-IIb + IIc were determined as “nondepressed” types, whereas EGC types 0-IIc, 0-III, 0-IIc + IIa, and type 0-IIc + IIb were classified as ‘depressed’ types. The histologic differentiation was classified into 2 groups according to dominant histology: a differentiated group with well-differentiated and moderately well-differentiated carcinomas, and an undifferentiated group with poorly differentiated and signet ring cell carcinomas.

Other pathologic findings included the number of resected LNs, Lauren classification of ulceration, lymphatic invasion, venous invasion, and tumor involvement at the resection margins (vertical and lateral margins).

### 2.4. Definition of local residual tumor

Follow-up surveillance endoscopy was performed within 3 months after ESD, and biopsy was routinely conducted in the ESD scar or any suspicious findings. Local residual tumor was defined as the presence of tumor cells in the previous ESD scar at the follow-up endoscopy.

### 2.5. Reassessment of the incidence of LNM and local residual tumors according to ESD indications

The indications for ESD were reassessed, and applicability was evaluated by comparing the incidence between our outcomes and Japanese Gastric Cancer Treatment Guidelines 2021 (ver. 6) outlined by the Japanese Gastric Cancer Association.^[7]^ The incidence of LNM and local residual cancer was investigated according to the histologic differentiation, tumor depth, presence of ulceration, and size from the pathological results of surgical specimens after additional gastrectomy (Table 1). The histopathologic characteristics of pre-ESD endoscopic forceps biopsy, ESD specimen, and surgical specimen of the patients with LNM and local residual cancer were analyzed (Table 2). The items examined on pre-ESD endoscopy included pre-ESD diagnosis, presence of ulceration, tumor size and location, histologic differentiation, and gross type. Tumor size, histologic differentiation, tumor invasion depth, presence of lymphatic invasion, and vertical and lateral margins in ESD specimens were investigated after ESD. After additional gastrectomy, presence of LNM and local residual cancer in surgical specimens and the number of harvested and positive LNs were included.

**Table 1:** Reassessment of the incidence of lymph node metastasis and local residual tumor according to the ESD indication criteria.

		No residual tumor (n = 113)	LN metastasis (n = 13)	Local residual tumor (n = 9)
Absolute and expanded indication of ESD				
Differentiated	pT1a (M), ULO	18 (3 HGD, 4 atypical)	1 (4.8%) (1 HGD)	2 (9.5%)
	pT1a (M), UL1, ≤3 cm	1	0	0
	pT1b (SM1), ≤3 cm	47 (13 HGD, 5 atypical)	3* (5.8%) (1 HGD, 2 atypical)	3* (5.8%) (1 HGD, 1 atypical)
Undifferentiated	pT1a, ULO ≤ 2 cm	7 (4 atypical)	0	1 (12.5%) (1 atypical)
Beyond indication of ESD				
Differentiated	pT1b (SM2)	36 (7 HDG, 7 atypical)	8 (17%) (2 HGD, 2 atypical)	3 (6.4%) (1 atypical)
Undifferentiated	>2 cm	1 (1 atypical)	0	0
	pT1b (SM1)	3	0	0
	pT1b (SM2)	0	1 (100%)	0

ESD = endoscopic submucosal dissection, HGD = high-grade dysplasia, LN = lymph node, M = mucosa, SM1 = minute submucosal invasion (<500 μm from the muscularis mucosae), SM2 = deep submucosal invasion (≥500 μm from the muscularis mucosae), UL0 = absence of ulceration, UL1 = presence of ulceration.

* One of them had both LN metastasis and local residual tumor.

**Table 2:** Detailed clinicopathologic characteristics with lymph node metastasis and local residual tumor in patients with non-curative ESD.

	Pre-ESD Diagnosis	Sex/age	Location	Size (pre-ESD)	Size (post-ESD)	Gross*	Diff.	Depth	Ly	Vertical margin	Lateral margin	Residual tumor	LN meta	Harvest LN	Positive LN	Recur
1	Atypical	M/66	LB	15	19	III	D	SM1	+	−	−	+	+	34	1	**+**
2	Atypical	M/61	LB	10	13	IIc	D	SM1	−	+	−	−	+	19	2	−
3	Atypical	M/64	LB	30	34	IIa + IIc	D	SM2	+	+	+	−	+	42	1	−
4	Atypical	F/59	MB	35	22	IIc + IIb	D	SM2	−	−	−	−	+	51	1	−
5	Atypical	F/66	LB	15	12	IIa + IIc	D	SM2	+	+	−	+	−	35	0	−
6	Atypical	M/47	LB	15	22	IIa	U	M	−	−	−	+	−	52	0	
7	HGD	F/78	LB	20	20	IIa + IIb	D	M	+	−	−	−	+	32	1	−
8	HGD	M/61	MB	15	30	IIa + IIb	D	SM1	−	−	−	−	+	45	3	−
9	HGD	M/65	LB	20	3	IIc	D	SM2	+	−	−	−	+	38	1	−
10	HGD	F/52	LB	15	15	IIa	D	SM2	−	−	−	−	+	31	1	−
11	HGD	M/45	LB	30	5	IIb + IIc	D	SM1	−	−	−	+	−	33	0	−
12	Adenoca.	M/74	LB	20	24	I	D	SM2	+	−	−	−	+	43	1	−
13	Adenoca.	F/74	LB	15	15	IIc	U	SM2	+	−	−	−	+	66	3	−
14	Adenoca.	M/68	UB	20	18	IIa + IIc	D	SM2	+	+	−	−	+	24	1	−
15	Adenoca.	M/60	LB	30	20	I	D	SM2	+	+	−	−	+	50	2	−
16	Adenoca.	M/60	UB	20	14	IIa	D	SM2	−	+	−	−	+	31	1	−
17	Adenoca.	M/54	MB	20	20	IIb + IIa	D	M	−	−	−	+	−	20	0	−
18	Adenoca.	M/60	MB	15	24	IIb	D	M	−	−	+	+	−	32	0	−
19	Adenoca.	M/47	LB	20	29	IIa + IIc	D	SM1	−	−	−	+	−	29	0	−
20	Adenoca.	M/73	LB	20	22	IIa	D	SM2	−	−	−	+	−	18	0	−
21	Adenoca.	F/87	UB	10	9	IIa	D	SM2	−	−	−	+	−	13	0	−

Adenoca = adenocarcinoma, D = differentiated group, Diff = differentiation, ESD = endoscopic submucosal dissection, F = female, HGD = high-grade dysplasis, LB = lower body, LN = lymph node, Ly = lymphatic invasion, m = male, M = mucosa, MB = middle body, SM1 = minute submucosal invasion (<500 μm from the muscularis mucosae), SM2 = deep submucosal invasion (≥500 μm from the muscularis mucosae), U = undifferentiated group, UB = upper body.

* According to the Japanese Classification of Gastric Carcinoma.

### 2.6. Statistical analysis

Student *t* test was used to compare continuous variables, and Pearson chi-squared test and Fisher exact test were used to compare categorical variables. A multivariate analysis was performed on significant variables in the univariate analysis to identify risk factors related to LNM and local residual tumors. All statistical analyses were performed using the SPSS version 26.0 (SPSS Inc., Chicago, IL), and significance was set at *P* < .05.

## 3. Results

**Figure 2.**
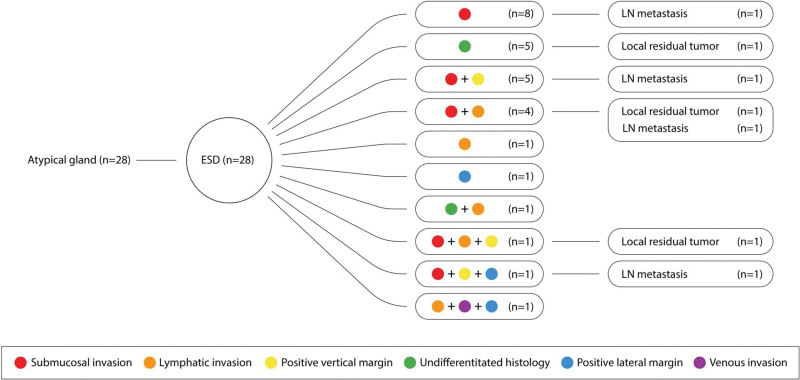
Reasons for requesting an additional gastrectomy and incidence of lymph node metastasis and local residual tumor in patients with atypical glands. LN = lymph node.

**Figure 3.**
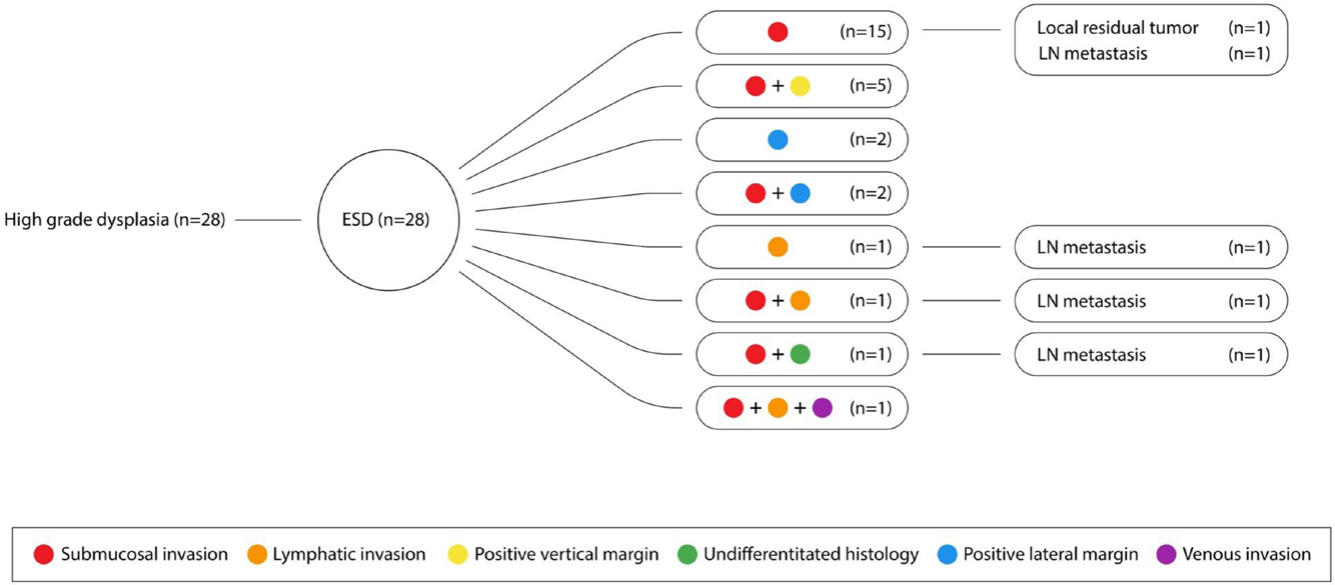
Reasons for requesting an additional gastrectomy and incidence of lymph node metastasis and local residual tumor in patients with high-grade dysplasia. LN = lymph node.

Based on the pathological results of ESD specimens, all patients with non-curative ESD were transferred to the surgical department for additional gastrectomy with LN dissection for one or more of the following reasons: deep submucosal invasion, lymphatic invasion, positive vertical margin, undifferentiated histology, positive lateral margin, and venous invasion (Table 3, Figs. 2 and 3). Of the 134 patients with EGC in the final pathology of ESD specimens, 56 patients (56/134, 41.8%) underwent staging ESD for a diagnostic approach, of whom 28 patients were diagnosed with atypical glands and the other 28 with high-grade dysplasia (HGD) prior to ESD. The remaining 78 of the 134 patients (78/134, 58.2%) were diagnosed with adenocarcinoma and received ESD for therapeutic purposes (Fig. 1). The mean interval between ESD and additional gastrectomy was 43.7 ± 24.8 days, and the most common operation method was totally laparoscopic distal gastrectomy, which was performed in 81 patients (81/143, 56.6%) (Table 4). No surgery-related mortality was recorded, although 5 patients (5/143, 3.5%) had postoperative complications according to Clavien classification (Table 5). Regarding surgical specimen, 13 patients (13/134, 9.7%) had LNM, and 9 patients (9/134, 6.7%) had local residual tumors, and among them, 1 patient with LNM presented simultaneously with local residual cancer cells at the ESD margin of the surgical specimen (Table 3, Fig. 1).

**Table 3:** Reasons for requesting an additional gastrectomy with lymph node dissection in patients with non-curative endoscopic submucosal dissection.

Request for operation	Number	Residual tumor (%)	Lymph node metastasis (%)	Recurrence
Submucosal invasion	56	4 (7.1)	2 (3.6)	0
Submucosal invasion + lymphatic invasion	18	1 (5.6)	3 (16.7)	0
Submucosal invasion + positive vertical margin	15	0 (0)	2 (13.3)	0
Undifferentiated histology	13	2 (15.4)	0 (0)	0
Lymphatic invasion	8	0 (0)	1 (12.5)	0
Positive lateral margin	6	1 (16.7)	0 (0)	0
Submucosal invasion + positive lateral margin	3	0 (0)	1 (33.3)	0
Submucosal invasion + venous invasion	3	0 (0)	0 (0)	0
Undifferentiated histology + lymphatic invasion	2	0 (0)	0 (0)	0
Submucosal invasion + undifferentiated histology	2	0 (0)	1 (50)	0
Submucosal invasion + positive vertical margin + lymphatic invasion	2	1 (50)	1 (50)	1 (multiple liver metastasis)
Submucosal invasion + lymphatic invasion + venous invasion	2	0 (0)	0 (0)	0
Submucosal invasion + positive vertical margin + lymphatic invasion + venous invasion	1	0 (0)	1 (100)	0
Submucosal invasion + positive vertical margin + positive lateral margin	1	0 (0)	1 (100)	0
Positive lateral margin + lymphatic invasion + venous invasion	1	0 (0)	0 (0)	0
Venous invasion	1	0 (0)	0 (0)	0

**Table 4:** Clinicopathologic characteristics and relationships with lymph node metastasis and local residual tumor in patients with non-curative ESD.

Variables	LN metastasis		*P*-value	Local residual tumor		*P*-value
	Presence (n = 13)	Absence (n = 113)		Presence (n = 9)	Absence (n = 113)	
Sex (male/female)	9/4	85/28	.737	7/2	85/28	.864
Age (years)	64.69 ± 7.23	64.45 ± 9.67	.931	60.56 ± 13.99	64.45 ± 9.67	.264
BMI (kg/m^2^)	24.69 ± 3.08	24.24 ± 3.13	.626	25.75 ± 2.96	24.24 ± 3.13	.167
Operation method (TLDG/LADG/STG/TLTG/TG/LAPG)	5/4/2/1/1/0	71/19/4/2/9/8	.132	5/3/0/0/1/0	71/19/4/2/9/8	.769
Operative time (min)	189 ± 48	185 ± 36	.721	176 ± 34	185 ± 36	.46
Blood loss (mL)	67.69 ± 74.63	68.05 ± 58.71	.984	65.56 ± 46.13	68.05 ± 58.71	.882
Tumor size (mm)	19.0 ± 7.81	17.46 ± 8.45	.534	18 ± 7.75	17.46 ± 8.45	.854
Tumor depth (M/SM1/SM2)	1/3/9	27/50/36	.028	3/3/3	27/50/36	.765
Longitudinal lesion (UB/MB/LB)	2/2/9	18/7/88	.474	1/2/6	18/7/88	.206
Circular lesion (LC/GC/AW/PW)	8/2/2/1	56/33/9/15	.541	3/2/2/2	56/33/9/15	.399
Ulceration (Presence/absence)	0/13	24/89	.127	4/5	24/89	.209
Gross type (nondepressed/depressed)	8/5	65/48	.781	8/1	65/48	.083
Differentiation (undifferentiated/differentiated)	2/11	11/102	.624	1/8	11/102	.619
Lauren classification (intestinal/diffuse/mixed)	13/0/0	102/8/3	.5	9/0/0	102/8/3	.618
Lymphatic invasion (Presence/absence)	8/5	24/89	.002	2/7	24/89	.945
Venous invasion (Presence/absence)	1/12	7/106	.593	9/0	7/106	.442
Vertical margin (Positive/negative)	5/8	15/98	.019	1/8	15/98	.853
Lateral margin (Positive/negative)	1/12	10/103	.89	1/8	10/103	.586
Hospital stay (days)	10.46 ± 7.57	8.42 ± 2.72	.353	8.11 ± 2.09	8.42 ± 2.72	.74
Day of first flatus	3.0 ± 1.68	3.17 ± 1.22	.66	3.0 ± 1.0	3.17 ± 1.22	.694
Day of soft diet initiation	5.92 ± 2.21	5.81 ± 1.91	.837	5.22 ± 1.97	5.81 ± 1.91	.583

AW = anterior wall, BMI = body mass index, ESD = endoscopic submucosal dissection, GC = greater curvature, LADG = laparoscopic assisted distal gastrectomy, LAPG = laparoscopic assisted proximal gastrectomy, LB = lower body, LC = lesser curvature, LN = lymph node, M = mucosa, MB = middle body, PW = posterior wall, SM1 = minute submucosal invasion (<500 μm from the muscularis mucosae), SM2 = deep submucosal invasion (≥500 μm from the muscularis mucosae), STG = subtotal gastrectomy, TG = total gastrectomy, TLDG = totally laparoscopic distal gastrectomy, TLTG = totally laparoscopic total gastrectomy, UB = upper body.

**Table 5:** Postoperative complications according to Clavien classification after additional gastrectomy.

Complications (n = 5)	Number	Operation	Treatment	Hospital stay	Grade*
Intra-abdominal abscess	1	STG	Operation	35	IIIb
Duodenal stump leakage	1	TLDG	PTBD	15	IIIa
Intra-abdominal fluid collection	1	TLDG	Antibiotics	15	II
Intra-abdominal bleeding	1	LAPG	Transfusion	14	II
Pancreatitis	1	TG	Supportive care	14	I

LAPG = laparoscopic assisted proximal gastrectomy, PTBD = percutaneous transhepatic biliary drainage, STG = subtotal gastrectomy, TG = total gastrectomy, TLTG = totally laparoscopic total gastrectomy.

* Clavien–Dindo classification.

### 3.1. Histopathologic characteristics and relationships with LNM and local residual tumors

Table 4 summarizes the histopathologic characteristics of the 3 groups. Significant differences in tumor invasion depth (M/SM1/SM2, 1 [7.7 %]/3 [23.1%]/9 [69.2%], *P* = .028), presence of lymphatic invasion (presence/absence, 8 [61.5%]/5 [38.5%], *P* = .002), positive vertical margin (positive/negative, 5 [38.5%]/8 [61.5%], *P* = .019) were observed between the LNM group and no residual tumor group. Of the 13 patients with LNM, 1 patient had mucosal EGC (mEGC) (1/31, 3.2%), and 12 patients had submucosal EGC (smEGC) (12/103, 11.7%) (Tables 1 and 2). Multivariate logistic regression analysis revealed that deep submucosal invasion (SM2) (odds ratio [OR]: 4.99, 95% confidence intervals [CI]: 1.29–19.36, *P* = .02), presence of lymphatic invasion (OR: 8.32, 95% CI: 1.96–35.28, *P* = .004), and positive vertical margin (OR: 8.43, 95% CI: 1.64–43.21, *P* = .011) were significant risk factors for LNM (Table 6). However, no significant differences in the baseline demographics, including sex, age, and body mass index, or in surgery-related variables such as operation method, operative time, and blood loss were observed between the 2 groups. All other tumor-related pathological variables such as tumor size, lesion, ulceration, gross type, differentiation, Lauren classification, venous invasion, and lateral margin did not differ significantly between the 2 groups. No significant differences in postoperative recovery parameters including hospital stay, day of first flatus, and day of soft diet initiation were also observed between the 2 groups.

**Table 6:** Univariate and multivariate logistic regression analyses of factors associated with LN metastasis.

Characteristics	Univariate analysis			Multivariate analysis		
	*P*-value	95% CI	Odds ratio	*P*-value	95% CI	Odds ratio
Vertical margin (Positive vs negative)	.019	1.18–14.15	4.08	.011	1.64–43.21	8.43
Lymphatic invasion (Presence/absence)	.002	1.78–19.8	5.93	.004	1.96–35.28	8.32
Deep submucosal invasion (≥500 µm)	.013	1.39–16.67	4.81	.02	1.29–19.36	4.99

CI = confidence interval, LN = lymph node.

Meanwhile, 3 patients had mEGC (3/31, 9.7%) and 6 patients had smEGC (6/103, 5.8%) among the local residual tumor group, although this difference between the local residual tumor group and no residual tumor group was not significant. No significant differences in all variables were also observed between the 2 groups (Tables 1 and 2).

### 3.2. Reasons for requesting an additional gastrectomy with LN dissection

Tumor invasion into the submucosal layer was the most common reason for requesting additional gastrectomy with LN dissection (56/134, 41.8%), followed by undifferentiated histology (13/134, 9.7%) and lymphatic invasion (8/134, 6.0%) as a single cause. In addition, 82 patients were transferred with a single cause for surgery (82/134, 61.2%), whereas 52 patients had more than 2 causes (52/134, 38.8%) (Table 3, Figs. 2 and 3). Moreover, in 56 patients who underwent ESD staging for diagnosis, 28 patients were diagnosed with atypical glands by forceps biopsy prior to ESD, and the remaining 28 patients were diagnosed with HGD. Diagnostic discrepancies were observed between pre-ESD endoscopic biopsy and final ESD pathology results, ranging from precancerous lesion (atypical gland, HGD) to adenocarcinoma (Fig. 1). In patients with atypical glands, submucosal invasion was the most common indication for requesting surgery (8/28, 28.6%), and 13 patients had multiple indications (13/28, 46.4%) (Fig. 2). In patients with HGD, submucosal invasion was the most common reason for requiring surgery (15/28, 53.6%), and 10 patients were considered for surgery for multiple reasons (10/28, 35.7%) (Fig. 3).

### 3.3. Reassessment of the incidence of LNM and local residual tumors according to the ESD indication criteria

Table 2 summarizes the detailed pathological characteristics of the cases confirmed as LNM and local residual tumor after additional gastrectomy. According to the Japanese Gastric Cancer Treatment Guidelines 2021 (ver. 6) outlined by the Japanese Gastric Cancer Association, all 21 patients met the ESD indication criteria in the pre-ESD evaluations, although 9 patients (9/21, 42.9%) satisfied the indications in the final pathology report after additional gastrectomy.^[7]^

LNM was detected in 13 of 134 (9.7%) patients with EGC, and local residual tumor was identified in 9 patients with EGC (9/134, 6.7%). Among them, 4 patients (4/28, 14.3%) with atypical glands with diagnostic discrepancies were identified as having LNM, and 3 patients (3/28, 10.7%) had local residual tumors (one of them had both LNM and a local residual tumor). In patients with HGD, 4 patients (4/28, 14.3%) had LNM, and 1 patient (1/28, 3.6%) had a local residual tumor (Figs. 2 and 3, Tables 1 and 2).

Their detailed incidence based on the indications such as histologic differentiation, tumor depth, ulceration, and size are presented in Tables 1 and 2.

Patients with LNM meeting the ESD indications are as follows: 1 case of differentiated mucosal cancer of any size without ulcer (1/21 [4.8%], 1 HGD); and cases of differentiated submucosal mucosal cancer (SM1), ≤3 cm (3/52 [5.8%], 1 HGD 1 and 2 atypical gland) (1 patient with LNM presented simultaneously with a local residual cancer). Patients with local residual tumors meeting ESD indications were the following: cases with differentiated mucosal cancer of any size without ulcer (2/21 [9.5%], 2 adenocarcinoma) and cases with differentiated submucosal mucosal cancer (SM1), ≤3 cm (3/52 [5.8%], 1 HGD, 1 atypical gland, and 1 adenocarcinoma) (1 patient with LNM presented with concomitant local residual cancer), and 1 case of undifferentiated mucosal cancer ≤2 cm without ulcer (1/8, [12.5%], 1 atypical gland).

## 4. Discussion

This study was originally designed to identify clinicopathologic features and risk factors in patients with non-curative ESD, and the analysis of these features revealed several new meaningful findings. All the patients fulfilled the criteria for ESD indications and underwent ESD for curative resection. Their pre-ESD assessments revealed a negligible risk of LNM, although owing to the potential risk factors for LNM and local residual tumors after ESD, they were referred for additional gastrectomy with lymphadenectomy.

Previously published studies in patients with non-curative ESD have primarily targeted 2 areas: efficacy of additional gastrectomy and long-term prognostic outcomes.^[14–19]^ However, considering that the patients with non-curative ESD already have some potential risks of LNM and local residual tumors, compared to previous studies, this study focused on the role of ESD in ambiguous histologic results in screening biopsy, applicability of ESD indications, and the actual factors of non-curative ESD that lead to additional gastrectomy.

Thus, the major issues of this study are that a significant proportion of indeterminate pathology demonstrated by endoscopic forceps biopsy was finally identified as adenocarcinoma after ESD. Moreover, even if the ESD indications were strictly met in the pre-ESD assessments, significant cases of LNM and local residual tumors in the surgical specimen of patients who underwent additional gastrectomy with lymphadenectomy could be present. In addition, the main factors that proved to be non-curative ESD were deep submucosal invasion and lymphatic invasion, which correlated with other factors and their combinations.

The findings and messages of this study are as follows.

The first is to re-evaluate the role of ESD for indeterminate pathology in endoscopic forceps biopsy.

In our study, 56 of 134 patients underwent ESD staging for a diagnostic approach because endoscopic forceps biopsy revealed a precancerous lesion (28 atypical glands and 28 HGD) despite endoscopic macroscopic findings suggesting malignant lesion, and they were diagnosed as adenocarcinoma in the final histology of the post-ESD specimen (Fig. 1). Compared to the rate of diagnoses before resection, the rate of diagnosed adenocarcinomas significantly increased after ESD. These diagnostic discrepancy rates have been reported to range from 18.7% to 44.5%, which may be attributed to the fact that endoscopic forceps biopsy may be less accurate because it does not completely sample the entire lesion but only partial lesions.^[9,10]^ It may lead to missed gastric cancer, delayed timing of the application of treatment, and increased treatment costs due to repeated endoscopic biopsies. Furthermore, in the analysis of LNM and residual cancer, 4 patients (4/28, 14.3%) with atypical gland, who had diagnostic discrepancies, were identified to have LNM, and 3 patients (3/28, 10.7%) had local residual tumor (one of them had both LNM and a local residual tumor). Among the patients with HGD, 4 patients (4/28, 14.3%) were identified as having LNM, and 1 patient (1/28, 3.6%) had a local residual tumor (Figs. 2 and 3, Tables 1 and 2). If ESD was not performed, and these lesions were considered mere borderline pathology, the opportunity to treat gastric cancer could be missed, which is an unsettling option, especially in regions with a high prevalence of gastric cancer such as Korea and Japan. ESD can be a viable alternative in this area of clinical uncertainty.

Therefore, when the endoscopic macroscopic findings are suspicious for a malignant lesion, although the pathology report from endoscopic forceps biopsy may be inconclusive, such as in this study for atypical glands or HGD, ESD should be actively performed for diagnostic and therapeutic purposes.

The second is a reanalysis of the ESD indication criteria between pre-ESD endoscopic assessments and pathological results of surgical specimens after additional gastrectomy.

The final pathology results revealed that the proportion of beyond indication cases was high at 38.8% (52/134, 9 HGD, 11 atypical glands) despite strict eligibility criteria in the pre-ESD assessments (Table 1). Thus, suitable candidates for ESD should be selected based on the pre-ESD endoscopic assessments. Although the final curability criteria for ESD depend on the pathological evaluation of the resected specimen after ESD, the results of which may not be consistent with the initial endoscopic findings. These differences suggest that additional surgery may have been warranted owing to the possibility of LNM and local residual cancer. In our study, 9 of them (9/52, [17.3%], 2 HGD, 2 atypical gland) were identified to have LNM, and 3 patients (3/52, [5.8%], 1 atypical gland) had local residual tumor (Table 1). This indicates a significant discrepancy in the indication criteria between the pre-ESD diagnosis and final pathological diagnosis, and this also implies that accurately predicting tumor size, histological type, depth of invasion, and presence of an ulcer for the indication criteria for ESD through pre-ESD assessments including endoscopic forceps biopsy is difficult.

Meanwhile, patients with LNM who met the ESD indications are as follows: 1 case of differentiated mucosal cancer of any size without ulcer (1/21 [4.8%], 1 HGD) and cases of differentiated submucosal mucosal cancer (SM1), ≤3 cm with the presence of ulceration (3/52 [5.8%], 1 HGD, 2 atypical glands) (1 patient with LNM presented simultaneously with local residual cancer). Patients with local residual tumors who met the ESD indications comprised the following: cases with differentiated mucosal cancer of any size without ulcer (2/21 [9.5%], 2 adenocarcinoma) and cases of differentiated submucosal mucosal cancer (SM1), ≤3 cm with the presence of ulceration (3/52 [5.8%], 1 HGD, 1 atypical gland, and 1 adenocarcinoma) (1 patient with LNM presented with concomitant local residual cancer), and 1 case of undifferentiated mucosal cancer ≤2 cm without ulcer (1/8, [12.5%], 1 atypical gland) (Table 1). Gotoda et al have reported that no cases of LNM were detected in any cases of differentiated mucosal cancer without ulcer regardless of tumor size (0/929, 0%), and this study was pivotal in establishing the current criteria for ESD indications.^[20]^

The reported incidence of LNM was 0.2% in patients with absolute indications and 0.7% in patients with expanded indications in a systematic review and meta-analysis of 9798 patients.^[21]^ The Japanese Gastric Cancer Treatment Guidelines 2021 (ver. 6) has reported the incidence of LNM as follows: none (0/437, 0%) for differentiated mucosal cancer, ≤2 cm without ulcer; none (0/493, 0%) for differentiated mucosal cancer, >2 cm without ulcer; and none (0/488, 0%) for differentiated submucosal mucosal cancer (SM1), ≤3 cm.^[7]^

The results of these studies differed significantly from our results. This high proportion of LNM cases even within ESD indications in this study may be attributed to the application of non-curative ESD in patients who already have potential risks of LNM, whereas the aforementioned studies included all patients who underwent gastrectomy for EGC.

The results indicate a high incidence of LNM even in cases where the indication for ESD was met, and some lesions with LNM were precancerous such as atypical glands or HGD. Therefore, if ESD had not been performed, the patient would have missed the opportunity for curative treatment.

Therefore, the indications for the endoscopic treatment of EGC proposed by the Japanese Gastric Cancer Association remain controversial, and the factors that were referenced in these indications were also applied according to the histologic differentiation, presence or absence of ulceration, tumor size, and predicted tumor invasion depth in pre-ESD endoscopic assessments.^[7]^ Furthermore, these indications have been established and expanded based on their association with LNM risk analysis in numerous pathological findings obtained from the surgical specimens of patients with EGC who underwent gastrectomy. This indicates that the applied ESD indications for patients with EGC are conceptualized before the implementation of ESD, and the assessments of the practicality for treatment are pathological features after ESD. Therefore, accurately confirming exclusion from criteria by pre-ESD assessments is difficult, and even if pre-ESD assessments are identified within these strict ESD indications, cases where pathological findings in the ESD specimen prove to be non-curative ESD may still be possible.^[8]^ Therefore, our results suggest that the role of ESD for diagnostic purposes should be re-evaluated.

The third is to analyze the factors associated with non-curative ESD requiring additional gastrectomy.

Submucosal invasion, undifferentiated histology, and lymphatic invasion were the most common single causes of non-curative ESD in this study, whereas the remaining cases were caused by simultaneous multiple factors (Table 3). Previous studies have similarly reported that lymphatic or vascular invasion, SM2 invasion, large tumor size, and positive vertical margin were associated with non-curative ESD, whereas our study reveals that it was often a combination of factors.^[22,23]^ Of note, in patients who underwent additional gastrectomy with LN dissection for non-curative ESD, the independent risk factors for LNM were identified as deep submucosal invasion, lymphatic invasion, and positive vertical margin (Table 6). If positive vertical margin was considered to be in the same category as that of tumor invasion depth, it can finally be compressed into 2 categories: deep submucosal invasion and lymphatic invasion. In addition, many studies have reported deep submucosal invasion and lymphatic invasion as risk factors for LNM, which is consistent in the present study.^[5,12,24]^ By contrast, lymphatic invasion can only be confirmed by final pathology results after ESD or gastrectomy, whereas in the case of submucosal invasion, the depth of tumor involvement can be assessed to some extent before these procedures.^[25,26]^

Therefore, in this study, deep submucosal invasion (>500 µm, SM2) was a major criterion for non-curative ESD and an independent risk factor for LNM. Thus, accurately estimating the depth of invasion in the pre-ESD assessment is important. Although this is a challenging task, it is essential for proper diagnosis and treatment planning.

Certain endoscopic features have been reported to be associated with SM invasion, including subepithelial tumor-like margin elevation, fusion of convergent folds, irregular nodular/depressed surfaces, and SM fibrosis; however, interobserver variability may be present in the identification of submucosal invasion by endoscopy.^[27]^ Furthermore, the accuracy of predicting the depth of tumor invasion with conventional endoscopy prior to ESD has been reported to be low (63–74%).^[22,28]^ Several authors have reported on the accuracy of EUS in assessing the depth of invasion in the pre-ESD assessments. However, the accuracy was only 70% to 76%, and the rate of overestimation of the depth of invasion was 18% to 42%.^[29,30]^ EUS is not suitable for all patients and may require additional time and financial resources. Thus, EUS has not been demonstrated to be superior to endoscopic evaluation in assessing the depth of invasion. Meanwhile, 2 systematic reviews have been conducted to evaluate the performance of artificial intelligence systems in estimating the depth of tumor invasion, with pooled sensitivity and specificity for predicting deep submucosal invasion ranging from 72% to 82% and 79% to 90%, respectively.^[31,32]^ Artificial intelligence systems have not yet been used in clinical practice because they are not more accurate than human experts at predicting tumor invasion depth. However, more research is needed in this area as this technology is expected to improve rapidly as research continues.

Therefore, the aforementioned findings ultimately suggest that ESD should be highly recommended for EGC treatment per se or preoperative assessment to accurately evaluate tumor invasion depth and presence of lymphatic involvement, and that the role of ESD in the design of EGC treatment strategies should be reconsidered.

Despite these valuable findings, this study has limitations. First, selection bias may have been present owing to the retrospective, single-center design and relatively small sample size of the study. Nevertheless, the data were prospectively collected from a database of patients at our institution. Second, EUS to assess tumor depth prior to ESD was not performed in all the patients, so the accuracy of this method could not be accurately compared with that of endoscopic evaluation. Lastly, we did not compare patients who were identified as having non-curative ESD and did not undergo additional gastrectomy as a control group. Although this study focused on patients who were referred to the Department of Surgery and we could not obtain data from patients who did not receive surgery, we were unable to evaluate how additional gastrectomy may have affected their prognosis. It is thus evident that further studies on the diagnostic role of ESD and the establishment of ESD indications are required in the future and this should be achieved by collecting data from non-curative and curative ESD patients from multiple centers.

## 5. Conclusion

When endoscopic assessments for gastric cancer screening yields equivocal or insufficient diagnostic results, ESD can be considered to play diagnostic and therapeutic roles in selecting optimal treatment for EGC because it provides more precise histologic information on the lesion, such as tumor invasion depth and lymphatic involvement, and offers a high curative resection rate through en-bloc resection.

## Author contributions

**Conceptualization:** Jae Hun Chung, Sun-Hwi Hwang, Si-Hak Lee.

**Data curation:** Dong Won Im, Jae Hun Chung.

**Formal analysis:** Dong Won Im, Jae Hun Chung.

**Investigation:** Si-Hak Lee.

**Resources:** Dong Won Im, Dae-Gon Ryu, Cheol Woong Choi, Su Jin Kim.

**Supervision:** Sun-Hwi Hwang, Si-Hak Lee.

**Validation:** Sun-Hwi Hwang.

**Visualization:** Dae-Gon Ryu, Cheol Woong Choi, Su Jin Kim.

**Writing – original draft:** Dong Won Im, Jae Hun Chung, Sun-Hwi Hwang, Si-Hak Lee.

**Writing – review & editing:** Si-Hak Lee.
